# Fast Flexible Transistors with a Nanotrench Structure

**DOI:** 10.1038/srep24771

**Published:** 2016-04-20

**Authors:** Jung-Hun Seo, Tao Ling, Shaoqin Gong, Weidong Zhou, Alice L. Ma, L. Jay Guo, Zhenqiang Ma

**Affiliations:** 1 Department of Electrical and Computer Engineering, University of Wisconsin–Madison, Madison, WI 53706, USA; 2 Department of Electrical Engineering and Computer Science, University of Michigan–Ann Arbor, Ann Arbor, MI 48109, USA; 3 Department of Biomedical Engineering and Wisconsin Institute for Discovery, University of Wisconsin–Madison, Madison, WI 53706, USA; 4 Department of Electrical Engineering, University of Texas at Arlington, Arlington, TX 76019, USA; 5 University of California - Berkeley, Berkeley, CA 94720, USA

## Abstract

The simplification of fabrication processes that can define very fine patterns for large-area flexible radio-frequency (RF) applications is very desirable because it is generally very challenging to realize submicron scale patterns on flexible substrates. Conventional nanoscale patterning methods, such as e-beam lithography, cannot be easily applied to such applications. On the other hand, recent advances in nanoimprinting lithography (NIL) may enable the fabrication of large-area nanoelectronics, especially flexible RF electronics with finely defined patterns, thereby significantly broadening RF applications. Here we report a generic strategy for fabricating high-performance flexible Si nanomembrane (NM)-based RF thin-film transistors (TFTs), capable of over 100 GHz operation in theory, with NIL patterned deep-submicron-scale channel lengths. A unique 3-dimensional etched-trench-channel configuration was used to allow for TFT fabrication compatible with flexible substrates. Optimal device parameters were obtained through device simulation to understand the underlying device physics and to enhance device controllability. Experimentally, a record-breaking 38 GHz maximum oscillation frequency *f*_max_ value has been successfully demonstrated from TFTs with a 2 μm gate length built with flexible Si NM on plastic substrates.

In recent years, flexible electronics have gained popularity with various applications ranging from flexible displays, wearable electronics and identification tags, biomedical devices, to structural health monitoring^1^,^2^,^3^,^4^,^5^,^6^. Many flexible electronics applications generally do not require the use of very high speed devices, but the flexibility of the electronics is of critical importance. Typically, the low speed flexible electronics are based on organic or low temperature deposition-compatible amorphous semiconductor (e.g., a-Si) or metal oxide materials, which can be processed with large area printing, coating, and deposition techniques^5^,^6^,^7^. On the other hand, radio-frequency (RF) capable flexible transistors, due to their wider signal handling capability, can extend flexible electronics applications toward wireless data transmission and wireless power transfer, or allow circuits to operate with much lower power consumption. The main challenges in the pursuit of RF flexible electronics included: (1) a lack of materials with sufficient mobility and simultaneous mechanical flexibility, and (2) difficulties in defining a fine channel region using a scalable fabrication process. Some solutions have been found to overcome the first challenge over the past decade. Flexible single crystalline semiconductor nanomembranes (NM) have adequately fulfilled the desired requirements^8^. However, patterning deep submicron scale features on the nanomembranes on flexible substrates using conventional fine lithography techniques^9^,^10^,^11^ has been very challenging due to the difficulties encountered in the fabrication process, such as the diffraction of exposed light on the plastic substrate and particularly the thermal plasticity of the flexible substrates under even moderate temperatures that are essential for photolithography. In addition, the conventional selective doping process via ion implantation and thermal diffusion can lead to unwanted short circuit due to easy merging between source and drain n+ wells (as shown in Fig. 1(b2))^9^,^10^,^11^,^12^. Such challenges associated with the conventional field effect transistor structure and its standard processes become more critical when dimensions are scaled down, thereby limiting the performance of flexible electronics (Fig. 1b). As of today, the smallest channel length of flexible transistors made on plastic substrates using the semiconductor nanomembranes is about 1 μm^9^,^10^,^11^.

To address these challenges and enable large-scale fabrication of high-performance RF flexible electronics, we have designed and demonstrated high-performance flexible TFTs on a polyethylene terephthalate (PET) substrate (Fig. 1(a)). The flexible RF TFTs were fabricated on flexible Si NM employing a nano trench structure produced via nano-imprinting lithography (NIL) technology and were transfer printed onto a PET substrate. We employed Si NMs created from a silicon-on-insulator (SOI) wafer, instead of organic and amorphous semiconductor materials, in order to achieve high enough mobility for TFTs to operate in the RF regime. Si NMs have been widely used in versatile high performance flexible electronics and optoelectronics applications^8^,^9^,^10^,^11^,^12^,^13^,^14^,^15^,^16^,^17^, because they not only have good flexibility and durability^17^, similar to other organic materials, but also have superior charge carrier mobility and saturation velocity^8^. One key feature of this novel TFT device structure is the nano trench formed in the Si NM via NIL, which is used to define a very small effective channel (as narrow as 100 nm)^18^,^19^. Unlike the previous selective doping approach where the smallest feature size is limited by doping process control^9^,^10^,^11^,^12^, the physical feature size of NIL sets the limit and it is completely independent of the doping conditions for the source and drain regions. Furthermore, the effective channel length (L_ch_) is not affected by the actual length of the gate electrode (L_g_). Namely, unlike the conventional methods, a deep submicron effective channel can be formed without the need of forming a nanoscale gate electrode. In this work, a longer gate-length electrode was deliberately used for easing and for reducing the cost of lithography. Such a structural advantage also offers a unique current path along with the trench (as marked in red in Fig. 1(a3)), which circumvents several physical issues when the effective channel length is reduced to the nanoscale, such as the short channel effect.

The comparison of cross sections between nano trench flexible RF TFTs and conventional TFTs are shown in Fig. 1(a,b). The detailed illustration of the conceptual geometrical difference in the effective channel region between conventional TFTs and nano trench TFTs is displayed in Fig. S4. When the effective channel length in conventional TFTs becomes narrower, the device suffers several physical issues. For example, when the effective channel length becomes nanoscale, a short channel effect is seen. However, the present nano trench TFTs not only offer structural advantages (the effective channel length is only decided by the length of trench), but also circumvent aforementioned issues. Our NIL defined trench TFTs have unique current flow path from source to drain. Unlike conventional TFTs, in which current flows from the source to the drain in a direction parallel with the channel (2-dimensional: 2D), the current in our TFTs initially flows upward from source through channel region and then downward into drain region (3-dimensional: 3D). Thus, the path of the current runs both perpendicular and parallel to the channel layer as it passes from the source to the drain under the gate dielectric layer (Note: the effective channels are marked with red in Fig. 1(a,b)). Current originates from the *n*+ source (for an NMOS device) in conventional TFTs, but our device used a partially *n*+ heavily doped layer in the p− Si NM layer as a current injection point. Since the n+ layer was defined by a NIL defined trench, current should flow near the trench and be controlled by the electric field of a gate metal.

Figure 2 shows the schematic illustration, cross section, and corresponding microscopic images for the fabrication process. Since our approach is geared toward the fabrication of large-area high performance flexible electronics, all of the device fabrication processes were designed to be carried out under low temperatures (lower than 150 °C) except for the first doping and recrystallization steps, which can be carried out in a blanket fashion before releasing Si NM from SOI^20^,^21^,^22^. The detailed processing conditions can be found in the Method section. Briefly, a lightly doped p–type SOI wafer with 270 nm thick Si template layer was implanted with phosphorus ions to make the surface (down to a depth of 180 nm) *n*+ doped, while the rest of the device layer (90 nm) remained lightly p− doped. Then nanoscale trenches were defined in the device layer by NIL followed by dry etching to separate and define a *n*+/*p*−/*n*+ current path from a drain to a source (Fig. 2(a)). Thereafter, the top Si device layer (i.e., the Si NM) was released and the source/drain electrodes were defined (Fig. 2(c)). The device was subsequently flip transferred onto an adhesive coated PET substrate (Fig. 2(d)). Since all of the layers were flipped, the source/drain electrodes were then positioned under the Si NM layer. The final fabrication steps involved additional dry etching to isolate/define the channel region and deposition of the gate dielectric layers and metal gate (Fig. 2(f)). The 2 μm length gate electrode that we used allowed easy alignment with the narrow trench (100–500 nm) during photolithography.

Two dimensional (2D) device simulation results under a bias condition (2 V to the gate and drain) using Silvaco^TM^ are shown in Fig. 3(a,b) to illustrate the path of the current flow. Figure 3(a) shows the simulated current density for trench depths of 200 nm, 220 nm and 250 nm, respectively. The red region represents a higher current density. It should be noted that the *n*+/p− junction in the Si NM was about 180 nm deep, as such, the remaining p− channel region was 70 nm thick for the 200 nm trench Si NM, while the remaining p− channel region was 20 nm thick for the 250 nm trench Si NM. The depth of the etched trench affected the efficiency of the current flow, as demonstrated by simulation with the 200, 220, and 250 nm trench depths and fixed trench width (i.e., L_ch_) of 100 nm. When the trench was 200 nm deep, a leakage current was observed near the upper part of the trench surface (Fig. 3(a)(i)). Although most of the current was drawn to the Si NM/oxide interface by the gate electric field, some of the current flowed through the trench surface without passing through the channel. When the trench was 220 nm deep, the leakage current began to decrease. When the trench reached 250 nm deep, the leakage current was well-suppressed (Fig. 3(a)(iii)). Generally, a deep trench, which provides a thin channel region, reduces leakage current because the current is more effectively drawn to the semiconductor/gate dielectric interface by the gate electric field. Thus, it is critical to etch a trench with precise depth to enhance the gate controllability and minimize the leakage current. Figure 3(b) shows the simulated current density near the channel region in devices with trench width (L_ch_) of 100 nm, 200 nm and 500 nm, respectively, with the trench depth fixed at 200 nm, which was the depth exhibiting the worst case scenario as illustrated in Fig. 3(a). As the trench width (L_ch_) became wider, the leakage current became smaller with the majority of the current flowing through the channel region. It was also observed that the channel became relatively thinner as the width of the trench became wider. Overall, it was shown that TFTs with narrower and shallower trenches show more leakage current due to a thicker channel region and weaker field-effect controllability. Therefore, the dimensions of the trench can be optimized in order to provide transistor performance characteristics appropriate for the intended application of the devices.

A comparison of the measured transfer and output characteristics for devices with various trench widths (i.e., channel length, L_ch_: 100 nm, 200 nm, and 500 nm) is shown in Fig. 4. The gate length (L_g_) is 2 μm and the depth of trench for all fabricated TFTs were fixed to 2 μm and 200 nm, respectively. It is noted that the length of the channel region in the TFT is determined by the width of trench and, therefore, is not determined by the gate length (L_g_) as it is in a conventional field-effect transistor. Because the channel length (L_ch_) is independent of the gate length (L_g_), L_ch_ can be very short – much shorter than L_g_, as shown in the simulated results (Fig. 3). As shown in Fig. 4(a), the output curve (*I*_*ds*_ − *V*_*ds*_) for a TFT with a 100 nm trench width showed poor saturation, which is attributed to the inaccurate trench etching to the desired depth as expected by the simulation in a Fig. 3(a). As the trench width increased to 200 nm and 500 nm (Fig. 4(b,c)), the drain currents were more saturated. The transfer curves for all three cases, with *V*_*ds*_ = 0.1 V, are plotted in Fig. 4(d). The peak transconductance of the devices slightly increased from 79 μS to 90 μS, as the trench was narrowed from 500 nm to 100 nm, which was ascribed to the concentrated conductivity of the stronger field-effect in the channel region. As the simulation result shown in Fig. 3(b), TFTs with 100 nm trench widths had a relatively short channel region with a graded current density distribution which means that the electron movement could be easily limited by such a drastic change in the field-effect. On the other hand, TFTs with a 500 nm trench width had a uniform current density distribution. This phenomenon also agreed well with the calculated field-effect mobility. The field-effect motilities for the TFTs with trench widths of 100, 200, and 500 nm were 155, 250, and 460 cm^2^/V·s, respectively, and were extracted according to the equation (1) 23,


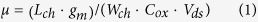


where *L*_*ch*_ and *W*_*ch*_ were the channel length and width, and *g*_m_ and *C*_*ox*_ were the transconductance and oxide capacitance, respectively. As the simulation results shown in Fig. 3(b), a narrower trench led to a higher inversion layer charge density. The low effective charge carrier mobility observed with the narrow trench TFTs are attributed primarily due to the low transconductance (poor gate controllability) and higher channel sheet charge density.

Deeper etching of the trench for the narrower width trenches should readily improve the gate controllability, as shown in Fig. 3, and thus improve the effective charge carrier mobility.

The subthreshold swing (*SS* = *d(V*_*gs*_)/*d*(*log*[*I*_*ds*_]) values of 330, 280, and 170 mV/dec were calculated from the linear portion of the *log*(*I*_*ds*_) versus *V*_*gs*_ plot from the TFTs with 100, 200, and 500 nm wide trenches, respectively. The relatively large subthreshold swings in these TFTs are attributed to the passivated channel surface. As the channel trench in the TFTs gets shorter, relatively more unpassivated surface exist which causes higher subthreshold swings. However, the subthreshold leakage is considered well-suppressed compared to other nanowire-based RF FETs^24^. Furthermore, the TFTs show no significant change in drain current after bending cycles of 20 times as shown in Fig. S6(c).

A microscope image of a bent array of TFTs and an array of ring oscillators on PET and that of a single 5-stage ring oscillator are shown in Fig. 4(e). TFTs with 200 nm wide trench (L_ch_) and 20 μm channel width were used to demonstrate 5-stage ring oscillator. Figure 4(f) shows the measured wave form at a supply voltage (*V*_*DD*_) of 2 V from one of the ring oscillators. The oscillation frequency and corresponding stage delay were 169 MHz and 0.59 ns, respectively.

Figure 5(a–c) present current gain (*H*_21_) and maximum stable/available gain (MSG/MAG, G_max_) derived from the measured scattering (S-) parameters at a *V*_*ds*_ of 1.5 V and a *V*_*g*_ of 0.6 V for TFTs with 100 nm and 200 nm wide trenches, and a *V*_*ds*_ of 1.2 V and a *V*_*g*_ of 0.6 V for a TFT with a 500 nm wide trench, respectively. The *f*_T_ and *f*_max_ were measured at 5 GHz and 38 GHz for a TFT with a 100 nm wide trench, 4.9 and 31 GHz for a TFT with a 200 nm wide trench, and 4.2 and 25 GHz for a TFT with a 500 nm wide trench. These results represent the highest speed of flexible TFTs made of Si. Regardless, these numbers do not imply the speed limit of the Si NM nano trench TFTs. As mentioned earlier, deeper etching of the narrower trenches (e.g., for the 100 nm case) will significantly improve the gate controllability of the channel and thus further greatly enhance both the *f*_T_ and *f*_max_ of the TFTs (see Figsure S5 for the speed predications using simulations under optimized dimensions). Figure 5(a–c) show that there was a reasonable agreement between the measured and simulated *f*_T_ and *f*_max_ values for the devices under the actually fabricated dimensions. The RF characteristics were further analyzed by employing a small-signal equivalent circuit model, the ADS2013 (Agilent Technology), to extract each parameter from the measured *S*-parameters at the bias conditions where the highest frequency responses were measured^25^ (Fig. 5(h)). The extracted figure-of-merit (FOM) values for various TFTs with different trench widths are summarized in Table 1. The extracted parasitic capacitance value of *C*_gs_ + *C*_gd_ obtained from the RF analysis was about 23 to 30 fF, which was comparable to that determined from the direct measurements of *f*_T_ and *g*_m_. The *f*_T_ value of ~5 GHz was extracted using the equation (2) 23,


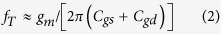


This measured value agrees well with the measured *f*_T_. As shown in Fig. 5(d), *f*_T_ increased with an increase of the drain biases. On the other hand, as shown in Fig. 5(e), *f*_max_ showed varied changes with gate biases, which is due to its monotonic dependence on *f*_T_ and other non-monotonic dependence on other device parameters. Figure 5(f,g) show the measured *f*_T_ and *f*_max_ variation trend as a function of tensile strain, which was consistent with that of the previous reports^10^,^26^. It was impossible to measure a frequency response under concave bending due to the large RF probe size in the setup. It is noted that the transistors remained intact and operational under high-strain conditions; a convex radius of curvature of 28.5 mm corresponded to an external strain of 0.55%. The TFTs also survived under hundreds times of bending under such a curvature, indicating the robustness of the 3-dimensional trench TFT structure. The detailed strain effects on the trenches in the TFTs were described in SI. In the 2-D simulations (Fig. S6(a,b)) for the 3-D trench TFTs, the trench was assumed to be infinitely long, which deviates from the actual situation where the trench region only accounts up a tiny fraction of the Si NM. As a result, the use of deep trench to form the unique 3-D TFTs does not risk the robustness of the TFTs due to the fractal near the trench^26^. Nevertheless, the mechanical robustness of TFTs could be further improved by applying additional layers, such as a polymer layer, to place the Si NM layer on the neutral plane^17^,^27^.

It should be noted that both the simulated and the demonstrated TFTs showed much higher *f*_max_ values than that of *f*_T_. Since *f*_T_ is mainly decided by the metal gate’s structure/dimensions, reducing the gate electrode dimension (gate length: L_g_) improves the *f*_T_. The nano trench TFT structure allows source and drain regions to be as close to each other as possible, unlike with the selective doping source/drain approach^9^,^10^,^11^,^12^. As a result, the access resistance has been substantially reduced^11^. For this reason, these TFTs exhibit record *f*_max_ values. Since transistors with high *f*_max_ values are more preferred for analog (e.g. RF) applications, the demonstrated nano trench approach implies great practical potential for fast flexible electronics.

Simulations with the typical scaling law of field effect transistors were carried out to further investigate the potential of the frequency characteristic of the device. As shown in Fig. S5, about 16 GHz *f*_T_ and 100 GHz *f*_max_ can be expected by simply applying a shorter gate electrode (1 μm or smaller). Of more importance, the *f*_T_ value can be further increased to 25 GHz by adapting ~ 45% intentional misalignment of the gate electrode to the drain. According to the simulated current density profile, such a performance enhancement is attributed to the re-distribution of current flow. As shown in Fig. S5, the best frequency performance was observed when the current density profile was nearly symmetric by 1 μm misalignment. Under this condition, the current flow was mostly concentrated on the channel region and gives the shortest current path from the source to the drain through the channel region. The higher current density at the channel region means the higher transconductance value (g_m_) and thus the intentional misalignment can directly improve the *f*_T_ and *f*_max_ values by the equations shown in the ref. 8. The simulation results indicated that the flexible Si NM TFTs with an optimal nano-channel defined by NIL have comparable performance with other types of flexible RF transistors (such as CNT or graphene FETs^24^,^28^), as well as conventional RF CMOS devices.

In conclusion, this study shows a viable approach for fabricating high performance flexible Si NM TFTs using the NIL technology. By applying the NIL process to define a deep-submicron channel, it is possible to realize flexible and RF-capable Si NM TFTs whose performance is comparable to the best existing flexible RF transistors made on rigid substrates or flexible TFTs made of nanowires. In addition, this unique 3-D device structure combined with the NIL technology may offer practical routes for mass production of high performance flexible RF active components/systems with nanoscale channels using a large area roll-to-roll NIL process at a lower cost. The new device structure and fabrication method are also easily applicable to III-V materials that have higher charge carrier motilities and higher overshoot velocities.

## Method

### Imprint mold fabrication

A photoresist (PMMA 950, Microchem) was spin-coated on the Si substrate with a thermally grown SiO_2_ layer, followed by electron-beam lithography to define the array of line patterns that will be the trench after the device fabrication. A 100 nm thick chrome (Cr) layer was deposited. Subsequently, the chrome layer in the unpatterned region was lifted off to yield a hard mask layer. The SiO_2_ layer was then carefully dry-etched using a reactive-ion etcher with a mixture of CF_4_ and O_2_ gases to remove the unmasked region. After removing the Cr patterns, the imprint mold was ready to be used.”

### Device fabrication

A silicon-on-insulator (SOI) wafer (from Soitec) with a lightly doped (4 × 10^15^ cm^−3^) *p*-type 270 nm top Si layer was doped uniformly (no patterning) with phosphorus via ion implantation. Prior to ion implantation, a 30 nm thick SiO_2_ was deposited as a screen layer by sputtering. The ion implantation was carried out at an energy level of 10 keV and a dose of 5 × 10^16^ atoms/cm^2^ at a 7° incident angle at room temperature. This was followed by a recrystallization process in a furnace at 900 °C for 20 min under nitrogen ambient. During the annealing process, a 180 nm deep *n*+ layer (at a peak doping level of 1 × 10^20^ cm^−3^) on the p− layer surface was formed due to phosphorus ion diffusion. As shown in Fig. S1, a test device was used to check the *p*− *n*+ junction diode, which showed a very good rectifying behavior in the implanted Si NM. A mr-I-7020E nanoimprinting photoresist (from Micro Resist Technology) was spun on an implanted SOI wafer, followed by thermal imprinting (Obducat AB NIL 2.5″ Nanoimprinter). Figure S2 provides the detailed imprinting conditions including pressure, temperature, and time. To make the detaching-step easy, the surface of the SiO_2_ imprinting mold was chemically treated with a self-assembled monolayer of a fluorosilane release agent (1H,1H,2H,2H-perfluorodecyl-trichlorosilane) using the chemical vapor deposition (CVD) method at 140 °C^29^. After completion of the imprinting step, and detachment of the mold from the SOI substrate, a weak oxygen plasma treatment was carried out using reactive ion etching (RIE, Unaxis 790) for de-scumming the remained photoresist (generally almost no photoresist is remained before de-scumming process) to fully expose the imprinting patterned regions on the SOI substrate as shown in Fig. S3(b). As shown in Fig. S3(c), the Si NMs were further etched by RIE with sulfur hexafluoride (SF_6_) gas under a low pressure (2 mtorr) to make 250 nm deep stiff sidewall trenches. Thereafter, using conventional photolithography, etching holes were defined and undercut on the imprinted top Si layer with diluted hydrofluoric acid (HF:H_2_O = 1:3 by volume) to release the Si NMs from the SOI handling substrate. After drying the Si NMs, the source/drain metal pads of Ti/Au (10 nm/150 nm) were deposited by an e-beam evaporator on top of the Si NM and then transferred together with the Si NMs onto an SU-8 coated PET substrate. Note that, after the transfer printing step, the source/drain metal pads and Si NMs were flipped and therefore, the source/drain metal pads were covered by the Si NMs. The Si NMs were patterned to define active regions using dry etching and, as a result, the source/drain metals underneath the Si NMs were exposed. Finally, gate dielectric and metal stacks (Al_2_O_3_: 100nm and Ti/Au: 10/200 nm) were deposited and lifted off. The I–V characteristics were obtained using an Agilent 4155B semiconductor parameter analyzer and the RF characteristics were obtained from the S-parameters measured using an Agilent E8364A performance network analyzer. The “open” and “short” features were used for a de-embedding procedure to obtain the intrinsic RF characteristics of the device. The detailed de-embedding procedure can be found elsewhere^10^,^25^.

## Additional Information

**How to cite this article**: Seo, J.-H. *et al.* Fast Flexible Transistors with a Nanotrench Structure. *Sci. Rep.* **6**, 24771; doi: 10.1038/srep24771 (2016).

## Supplementary Material

Supplementary Information

## Figures and Tables

**Figure 1:**
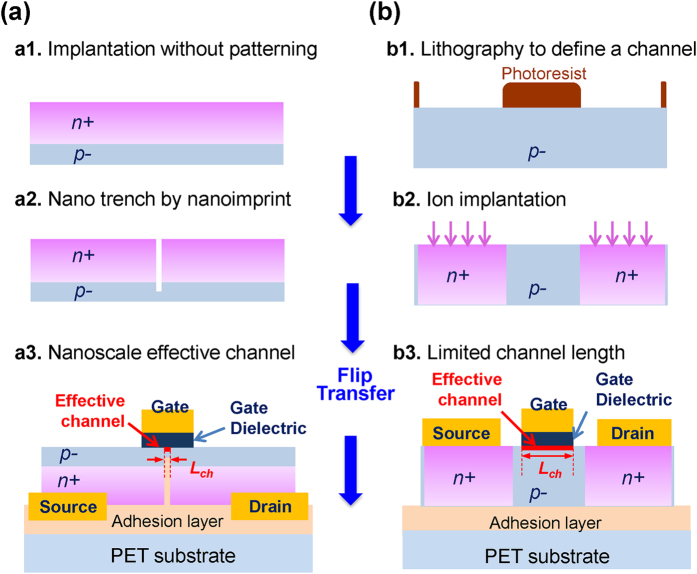
Comparison of the device structures (cross-sectional view) and fabrication processes between **(a)** 3-D nano trench Si NM flexible RF TFTs, and **(b)** conventional 2-D TFTs. The effective channel lengths L_ch_ are marked in red in (a3,b3). The smallest L_ch_ of the nano trench TFT can reach down to 50 nm via NIL and that of the conventional TFT can only reach down to about 1.5 μm. (a1) Blanket phosphorous ion implantation and thermal anneal. (a2) Nano trench formation via nanoimprint. (a3) Final structure of nano trench TFT where the channel length L_ch_ is defined by nanoimprint. (b1) Photolithography to define S/D regions for ion implantation. (b2) Selective ion implantation and thermal anneal. (b3) Final structure of conventional TFT where the channel length L_ch_ is limited by gate electrode and dopant out-diffusion during ion implantation and thermal anneal.

**Figure 2:**
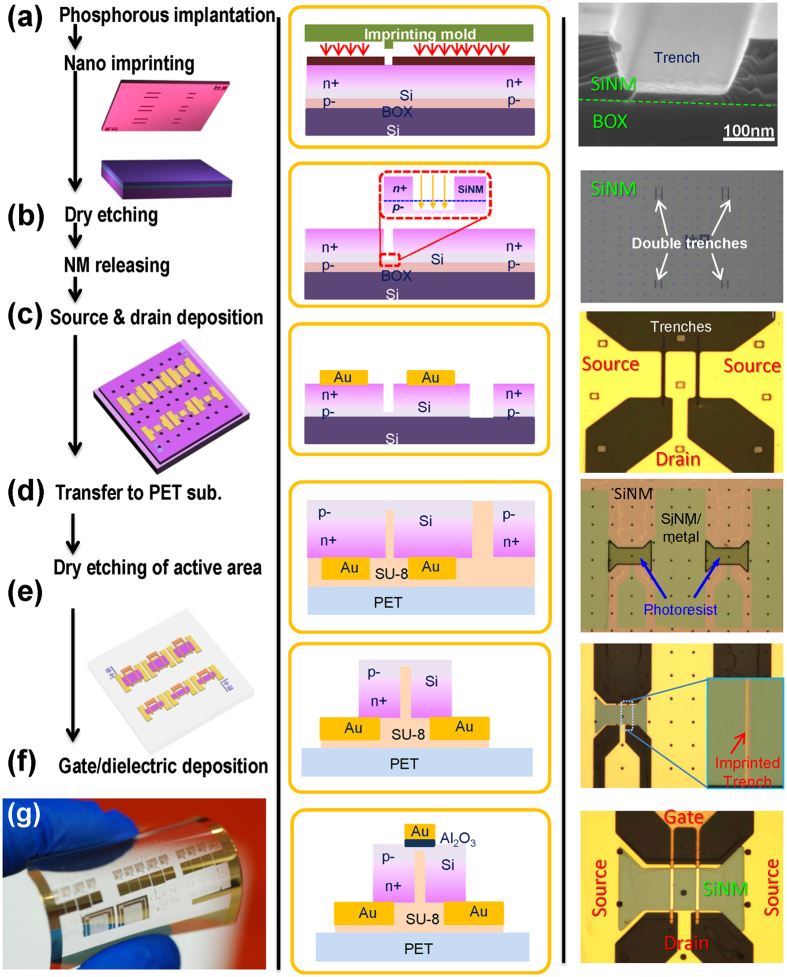
Fabrication process for nano trench Si NM flexible RF TFTs by NIL. Schematic illustration (left column), cross section structure (middle column), and corresponding microscopic images (right column) of nano trench Si NM flexible RF TFTs. **(a)** Defining a nano trench on a phosphorus implanted *p*− SOI substrate using NIL. **(b)** Dry etching to separate the *n*+ area in order to form a path of *n*+/*p*−/*n*+ from source to drain. **(c)** A partially completed TFT after undercutting the buried oxide to release the Si NM, which forms the active region, and forming the source and drain contacts. **(d)** Flip transfer of the Si NM with the source and drain electrodes onto an adhesive coated PET substrate. **(e)** Dry etching to define the perimeter of the active region. **(f)** Deposition of an Al_2_O_3_ gate dielectric layers and gold gate electrodes above the trench. (**g**) Optical image of arrays of the bent TFTs on a PET substrate.

**Figure 3:**
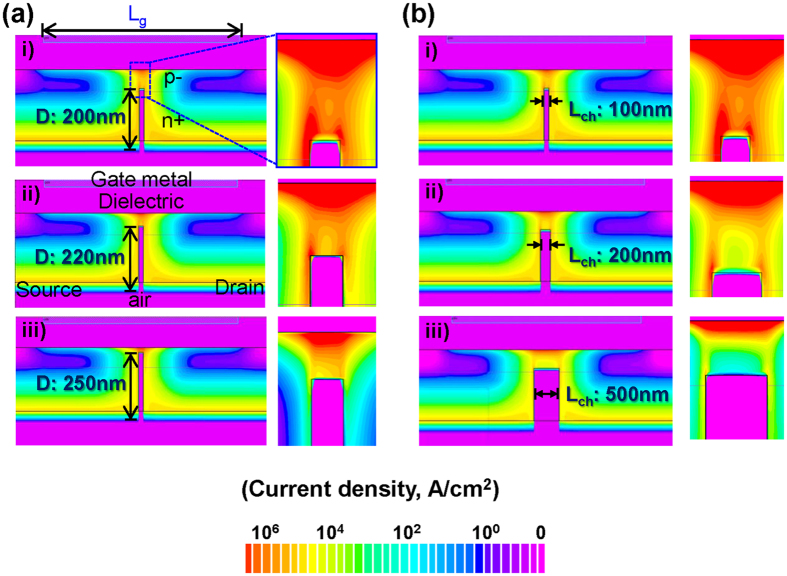
Simulated current density near the channel region for different depths and widths of the trench. The total thickness of the Si NM (*p*− layer plus *n*+ layer) is 270 nm. The thickness of the *n*+ layer is 180 nm and that of the *p*− layer thickness is 90 nm. For all the scenarios simulated, the metal gate length (L_g_) remains at 4 μm. **(a)** The trench width/channel length (L_ch_) is fixed at 100 nm. **(a)**(i) Simulated current density for a TFT with a 200 nm deep trench (20 nm of the trench depth extends into the *p*− layer: 70 nm *p*− layer remains as the active channel) revealing that the majority of the current flows through the trench surface and field-effect controllability is weak. (ii) At 220 nm deep (40 nm of the trench depth extends into the *p*− layer: 50 nm *p*− layer remains as the active channel), which is the middle value of the depth between the *n*+/*p*− interface and the top surface of Si NM, gate controllability is improved but a leakage current is still present through the trench. (iii) Simulated current density with the 250 nm deep trench (70 nm of the trench depth extends into the *p*− layer: 20 nm *p*− layer remains as the active channel) forms a very strong field-effected channel without a leakage current. **(b)** Dependence on the trench depth reduces as the width of the trench (L_ch_) becomes wider. The trench depth (D) is fixed at 200 nm. (i) The 100 nm wide and 200 nm deep trench shows a large leakage current near the trench surface. (ii) The 200 nm wide (L_ch_) trench significantly reduces the leakage current. (iii) The 500 nm wide (L_ch_) trench is completely free from the leakage current.

**Figure 4:**
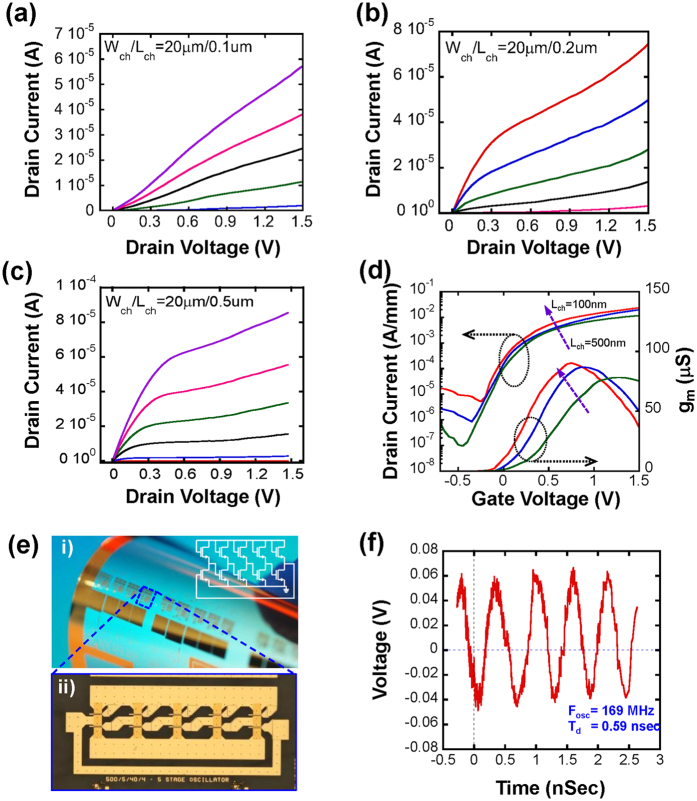
DC characteristics of the TFTs with various trench gaps/channel lengths (L_ch_). Drain current versus drain voltage, *I*_*d*_ − *V*_*ds*_, output curves are shown. All devices have 2 μm of gate length (L_g_) and biased with V_gs_ ranging from 0 V to 1.5 V with a 0.3 V step **(a)** Devices with a 100 nm gap and a channel width and length of 20 μm and 100 nm. **(b)** Devices with a 200 nm gap and a channel width and length of 20 μm and 200 nm. **(c)** Devices with a 500 nm gap and a channel width and length of 20 μm and 500 nm. (**d)** Merged drain current versus gate voltage, *I*_*d*_ − *V*_*gs*_, transfer curves and transconductance (g_m_) with *V*_*ds*_ = 0.1 V for these three devices. The two arrows show the directions of reducing L_ch_. **(e) i)** A microscope image of a bent array of TFTs and ring oscillators on a PET substrate. **ii)** A microscopic image of a single 5-stage ring oscillator under a flat condition**. (f)** Measured voltage–time characteristic of the 5-stage ring oscillator showing a *f*_osc_ of 165 MHz and a t_d_ of 0.59 nsec.

**Figure 5:**
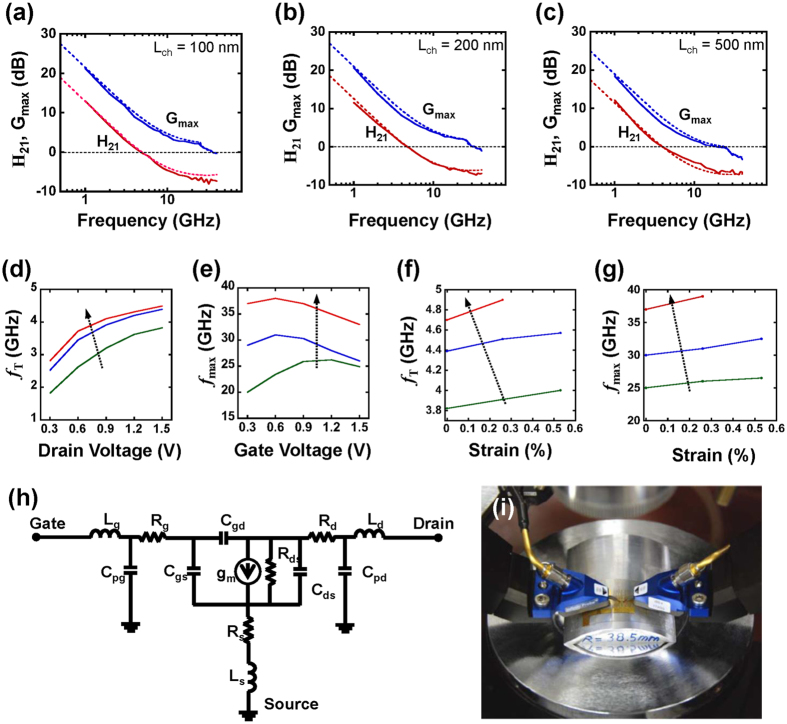
Measured (solid lines) and simulated (dashed lines) RF characteristics of the trench TFTs with various trench gaps/channel lengths (L_ch_). The gate length (L_g_) in all TFTs is 2 μm. Current gain (*H*_21_) and power gain (*G*_*max*_) as a function of the frequency of a Si NM TFT with a (**a**) 100 nm, (**b**) 200 nm, and (**c**) 500 nm wide trench (L_ch_). (**d**,**e**) *f*_T_ and *f*_max_ as a function of gate bias under a fixed drain bias (*V*_*ds*_ = 1.5 V) and as a function of drain bias under a fixed gate bias (*V*_*g*_ = 0.6 V for 100 nm and 200 nm TFTs and *V*_*g*_ = 1.2 V for 500 nm TFTs). (**f**,**g**) *f*_T_ and *f*_max_ as a function of bending induced external strain. (**h**) The small-signal equivalent circuit model used for TFT parameters extraction. (**i**) Image of bending setup for RF measurements.

**Table 1:** Comparison of the extracted device model parameters and figure-of-merit (FOM) values between TFTs with various trench widths, L_ch_.

Trench Width L_ch_(nm)	g_mo_(mS)	t (psec)	R_g_(Ω)	R_d_(Ω)	R_s_(Ω)	L_g_(nH)	L_d_(nH)	L_s_(nH)	C_gd_(pF)	C_gs_(pF)	C_ds_(pF)	R_ds_(Ω)	*f*_T_(GHz)	*f*_max_(GHz)
100	2.4	3.6	0.05	9.7	128	0.014	0.18	0	0.012	0.017	0.005	80	5	38
200	2.2	3.6	0.05	9.7	134	0.014	0.18	0	0.012	0.018	0.008	85	4.9	37
500	2.1	3.6	0.05	9.7	148	0.014	0.18	0	0.012	0.011	0.010	95	4.2	34
