# Supplementary material for: Fast Flexible Transistors with a Nanotrench Structure
Source: Sci Rep. 2016 Apr 20;6:24771. doi: 10.1038/srep24771 (PMC4837400; doi:10.1038/srep24771)
Supplement: Supplementary Information [file srep24771-s1.doc]

**Supplementary Information (SI)**

**Fast Flexible Transistors with a Nanotrench Structure**

Jung-Hun Seo^1^, Tao Ling^2^, Shaoqin Gong^3^, Weidong Zhou^4^, Alice L. Ma^5^, L. Jay Guo^2^, and Zhenqiang Ma^1*^

*^1^Department of Electrical and Computer Engineering, University of Wisconsin–Madison, Madison, WI 53706, USA*

*^2^Department of Electrical Engineering and Computer Science, University of Michigan–Ann Arbor, Ann Arbor, Michigan, USA*

*^3^Department of Biomedical Engineering, Wisconsin Institute for Discovery, and Materials Science Program, University of Wisconsin–Madison, Madison, WI 53706, USA*

*^4^Department of Electrical Engineering, University of Texas at Arlington, Arlington, TX 76019, USA*

*^5^University of California – Berkeley, Berkeley, CA 94720, USA*

^*^Authors to whom correspondence should be addressed. Electronic address: [mazq@engr.wisc.edu](mailto:mazq@engr.wisc.edu)

**Supplementary Information (S.I.)**

**Phosphorus doping for *n+* Layer Formation in a *p-* SOI Substrate**

Phosphorus doping was carefully simulated with Silvaco^TM^ using the Monte Carlo method. In this device, formation of a *n+/p-* junction with a depth of 180~200 nm is critical. Figure S1 (a)(i) shows the graphical distribution of implanted ions using 10 KeV of energy and 5×10^16^ atoms/cm^2^ of dose. Figure S1 (a)(ii) shows that a Gaussian distributed, heavily doped *n+* layer that was obtained after annealing at 900 ^o^C for 20 minutes in the furnace. Based on the simulation results, a *p-n* junction was expected to be formed at a depth of 180 nm. A *n+/p-* test diode was fabricated, as shown in a Figure S1 (b). 220~230 nm of Si was etched out of a 270 nm thick Si NM to expose the *p-* layer. The I-V characteristic showed good rectifying characteristics and confirms the formation of the *p-n* junction as shown in a Figure S1 (c).

**Figure S1. (a)** 2-D implantation simulations using the Monte Carlo method for deciding ion implantation dose and energy. (i) after ion-implantation and (ii) after thermal annealing. **(b)** Cross section of the *n+/p-* test diode. **(c)** Measured *I-V* characteristic from the *n+/p-* test diode shown in (b).

**Detailed NIL Conditions: Pressure and Temperature as a Function of Time**

For the thermal NIL processes, pressure and temperature are the two most important parameters that determine the quality of the NI patterns. A mr-I-7020E nanoimprinting photoresist (from Micro Resist Technology) was used and an NIL process was carried out using a thermal imprinting system (Obducat AB NIL 2.5” Nanoimprinter). It is key that the sample for the NIL process should be heated over the glass transition temperature (*T_g_*) before it is pressed. When detaching the sample, however, the temperature should be lowered first and then raised to *T_g_* before releasing the pressure. Adding several transient pressure and temperature steps also helps detach a mold from the substrate.

**Figure S2. (a)** Recorded pressure and temperature as a function of time in the NIL process. **(b) and (c)** Magnified temperature relationship when the stage pressure is applied and released.

**Trench Etching After NIL**

Trench etching is crucial for both *p-n* junction formation and inhibition of a leakage current. Trench etching starts with the removal of PR residue using weak O_2_ plasma, followed by Si etching. To maintain a straight side wall, we used a low pressure and high SF_6_/O_2_ ratio etching condition. Since devices are very sensitive to the trench depth, a precise etching condition was carefully tested and trench etching was stopped at about 250 nm in depth.

**Figure S3. Process for dry etching a trench. (a) and (b)** Removal of the photoresist residue after the NIL process by O_2_ plasma. **(c)** Dry etching down into the *p-* layer with a trench depth of ~250 nm. **(d)** Removing the NIL PR to finish the etching step.

**Conceptual Geometry Changes of Nano Trench Si NM Flexible RF TFTs**

Figure S4 shows the change of device geometry from the 2-D conventional Si NM TFT to the 3-D nano trench Si NM TFT. In the nano trench Si NM TFT, the effective channel length L_ch_ only formed on top of the trench, without being affected by the gate electrode (Figure S4 (d)). On the other hand, the effective channel length of conventional 2-D Si NM flexible RF TFTs solely depends on the length of the gate electrode.

**Figure S4. Conceptual geometrical changes from the 2-D conventional Si NM flexible RF TFTs to 3-D nano trench Si NM flexible RF TFTs: (a)** conventional TFT structure, **(b) and (c)** TFTs with a folded channel region to reduce the distance between source and drain regions, and **(d)** re-arranged structure of nano trench Si NM flexible RF TFTs.

**Simulated RF Characteristics for Scaled Nano Trench TFTs**

**Figure S5. Simulated current density and RF characteristics with (a)** the optimal structure, **(b)** gate electrode misaligned 0.45 μm left of center, and **(c)** gate electrode misaligned 0.45 μm right of center.

To investigate the potential RF performance of devices, RF characteristics of the trench TFTs were simulated by Silvaco^TM^ Atras. With an optimal device structure, a 100 nm wide by 250 nm deep trench with a 1 μm gate length without a gate to the source and drain overlap, *f*_T_ and *f*_max_ can ideally exceed 16 GHz and 100 GHz, respectively. The *f*_T_ and *f*_max_ are dependent on the device parameters such that the *f*_T_ and *f*_max_ can be lowered if one uses a wider gate length or a longer gate to the source and drain overlap distance. Notably, as can be seen in Figure S5 (b) and (c), intentional misalignment of the gate electrode can further enhance the RF characteristics. This improvement occurs because intentional misalignment corrects the asymmetrical current density distribution. When the gate electrode is perfectly aligned with the trench, the current distribution is not symmetric. Such an asymmetric distribution worsens when the gate electrode is misaligned to the left; but when it misaligned to the right (to the drain), the distribution of current density becomes symmetric. As a result, the total effective resistance from source to drain is reduced and this is attributed to the improvement of *f*_T_ and *f*_max_.

**Mechanical Simulations under Bending**

The 2-dimensional simulations were to investigate the strain effects on the etched nano trenches. The simulations imply the worst scenario where each trench width (W_ch_) is infinitely long and the trench extends to the full length of the PED substrate. It is noted that under the practical conditions, the trench width W_ch_ is much smaller than the PED substrate. Under the bending conditions where stress is applied in parallel to channel length direction, which is a worse situation than that if the stress is applied perpendicular to the channel length direction), the strain was concentrated on the trench area because the dimension of the trench was just 100 nm ~ 500 nm wide and the active channel is only 20−70 nm thick. Mechanical simulation by Ansys^TM^ also showed that the channel region (i.e., the trench area) had more strain than the peripheral region. This can further increase the mobility in Si NM.

**Figure S6. Simulated strain near the channel region with (a)** the compressive strain on the convex mold and **(b)** the tensile strain on the concave mold. **(c)** The bending reliability reflected by drain current vs. times of bending.
